# Metabolic Interaction Between Schwann Cells and Axons Under Physiological and Disease Conditions

**DOI:** 10.3389/fncel.2020.00148

**Published:** 2020-05-29

**Authors:** Filipa Bouçanova, Roman Chrast

**Affiliations:** ^1^Department of Neuroscience, KarolinskaInstitutet, Stockholm, Sweden; ^2^Department of Clinical Neuroscience, KarolinskaInstitutet, Stockholm, Sweden

**Keywords:** Schwann cell, axon, PNS, energy metabolism, peripheral neuropathy, ketogenic diet

## Abstract

Recent research into axon-glial interactions in the nervous system has made a compelling case that glial cells have a relevant role in the metabolic support of axons, and that, in the case of myelinating cells, this role is independent of myelination itself. In this mini-review article, we summarize some of those observations and focus on Schwann cells (SC), drawing parallels between glia of the central and peripheral nervous systems (PNS), pointing out limitations in current knowledge, and discussing its potential clinical relevance. First, we introduce SC, their development and main roles, and follow with an evolutionary perspective of glial metabolic function. Then we provide evidence of the myelin-independent aspects of axonal support and their coupling to neuronal metabolism. Finally, we address the opportunity to use SC-axon metabolic interactions as therapeutic targets to treat peripheral neuropathies.

## Introduction

From synaptic maturation to neurotransmitter recycling, it is now indisputable that glial function and dysfunction have a tremendous impact on the nervous system. Myelinating glia, in particular, have the power to either optimize neuronal signals or completely disrupt them, as shown by demyelinating conditions such as multiple sclerosis (Lemus et al., 2018) and Charcot-Marie-Tooth (CMT) disease (Brennan et al., 2015).

Schwann cells (SC) are the main glial cell type in the peripheral nervous system (PNS) of higher vertebrates. They arise from neural crest-derived precursors and mature into the main architects of peripheral nerves (Woodhoo and Sommer, 2008; Fledrich et al., 2019). Two types of mature SC can be observed: myelinating SC interact with large-caliber axons in a one-to-one relationship and wrap them in layers of lipid-rich compact membrane forming a myelin sheath; small-caliber axons are instead engulfed by non-myelinating SC (nmSC) in so-called Remak bundles. The molecular distinction between small (<1 μm) and large (>1 μm) axons comes from the level of neuregulin 1 type III expressed at the axonal surface (Michailov et al., 2004; Taveggia et al., 2005). Larger axons express more NRG1 and therefore can overcome a threshold in ErbB2/3-mediated signaling in the neighboring SC, triggering the myelination program. The process of caliber selection is called radial sorting and is essential for the proper maturation and long-term function of the PNS. Many demyelinating or dysmyelinating conditions can affect SC’s ability to establish and maintain myelin, with consequences for motor and sensory functions.

**Figure 1:**
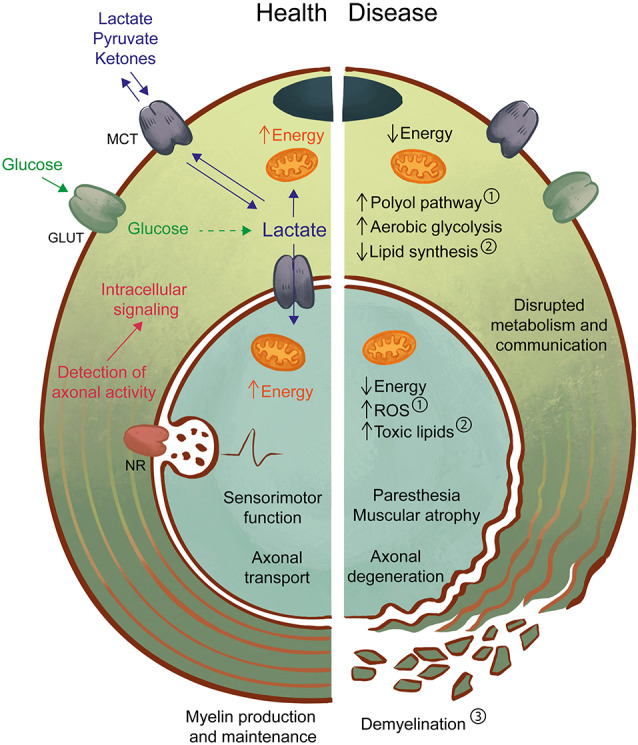
Axon-Schwann cell (SC) interactions in health and disease. In the healthy peripheral nervous system (PNS) (left), myelinating and non-myelinating SC support axonal function by providing physical/electrical insulation in the form of myelin as well as metabolic support. One possible mechanism may involve the detection of axonal activity by neurotransmitter receptors (NR) on SC, followed by intracellular signaling and activation of glucose metabolism. Lactate, a by-product of glycolysis can be an important source of energy for both SC and axons and is shuttled by monocarboxylate transporters (MCT). Axon-glia interactions likely contribute to the maintenance of normal sensorimotor function and transport of molecules and organelles along the axon. In the diseased PNS (right), disruption of SC-axon metabolic interactions may be both a cause and a consequence of pathology. For example, in diabetes ①, high levels of glucose lead to activation of the polyol pathway, mitochondrial dysfunction, and oxidative stress, which drive the development of neuropathic symptoms and axon degeneration. Also, experimental models of impaired SC metabolism, such as the LKB1 and TFAM mouse mutants, have demonstrated the effects of abnormal lipid production ② in axonal maintenance, with or without progressive changes to myelin ③. Primary myelin defects are also a driver of axonal loss, as reduced or defective myelin will impair axon impulse conduction and drive the development of neuropathy. Disruption of axon-glia metabolism and communication by any of these pathways can lead to axonal degeneration with abnormal sensory (e.g., paresthesia) and motor (e.g., muscular atrophy) function. Further studies of the SC-axon metabolic relationship are necessary to allow the prevention, early detection, and treatment of pathologies.

In addition to myelination, axonal support is now considered a distinct role of SC, and many researchers contributed to the identification of the mechanisms behind it. In this mini-review article, we will address the role of SC as metabolic supporters of axons in health and disease, summarized in Figure 1. We will draw parallels with glial cell types of the central nervous system (CNS) and point out some limitations in our current knowledge. Finally, we will conclude with a perspective on therapeutic applications of this metabolic interaction.

## Evolutionary Evidence of the Role of Axon-Ensheathing Cells in Metabolic Support

Axon-ensheathing cells are not only a feature of higher vertebrates but are also observed in invertebrates such as fruit flies (*Drosophila melanogaster*; Rodrigues et al., 2011) and jawless fish like the lamprey (*Petromyzon marinus*). The development of such cells precedes the emergence of structured myelin by several million years (Bullock et al., 1984) and suggests that myelin-independent axonal support might be the primary function of glia. In the lamprey peripheral lateral line nerve, glial cells similar to SC engulf multiple small-caliber axons in structures reminiscent of Remak bundles and sort out larger caliber axons (greater than 3 μm) into one-to-one structures (Weil et al., 2018). These cells express SoxE genes such as SoxE2 and SoxE3, which are equivalent to mammalian SC-specific Sox10 and Sox9, respectively, suggesting that they are orthologous to SC. Given the absence of myelin in these animals, it can be proposed that the main role of peripheral glial cells is in fact axonal support and that accelerated impulse propagation could be a beneficial side effect of the interaction.

It is well established that neurons utilize large amounts of energy to maintain membrane potential and ion gradients, and that they are particularly sensitive to oxidative stress (Chen et al., 2012). Evidence from *Drosophila* CNS shows that, in the eye, pigment cells (PC) produce lactate through glycolysis and ship it to neurons *via* monocarboxylate transporters (MCT; Liu et al., 2017). Under physiological conditions, neurons can use lactate for energy and lipid production. However, under stress conditions, such as high levels of reactive oxygen species, photoreceptors protect themselves by increasing the amount of lipids synthesized from lactate and shuttling them back to PCs *via* apolipoproteins, where they accumulate in lipid droplets. Disruption of neuronal MCTs prevents lactate import, reducing lipid synthesis and delaying neurodegeneration, but the loss of apolipoproteins prevents the transfer of lipids to glia and exacerbates neuronal loss.

In mammals, astrocytes are notorious for their storage of glycogen, which is necessary for long term memory (Suzuki et al., 2011), while oligodendrocytes, the myelinating cells of the CNS, can take up lipids from other cell types (Saher et al., 2005; Camargo et al., 2017) and N-acetyl aspartate from neurons to use for lipid synthesis (Chakraborty et al., 2001). Taken together, glycogen, lipid droplets, and myelin could be different types of energetic stores that neurons have outsourced to glial cells throughout evolution (Nave et al., 2017). SC, in particular, combine two of these strategies by producing myelin and glycogen granules (Brown et al., 2012). Contrary to unmyelinated C fibers, which quickly lose the ability to conduct action potentials *in vitro* unless exogenous substrates are available, glycogen breakdown and its conversion to lactate during periods of aglycemia may play a role in the support of the sustained activity of large, myelinated A fibers (Rich and Brown, 2018).

It is important to point out that some studies have provided conflicting evidence, suggesting that maybe not all neurons rely on their glial neighbors for energetic support (Chamberlain and Sheng, 2019). Therefore, additional studies must be made to further clarify the relationship between axons and glia.

## Indications That Glial Support of Axons Is Functionally Distinct From Myelination

Evidence from the mammalian CNS further indicates that myelination and axonal support are two independent functions. Genetic disturbances in oligodendrocytes, such as the PLP1 null mouse (Klugmann et al., 1997; Griffiths et al., 1998) can lead to neurodegeneration without prior de/dysmyelination. These mice assemble normal myelin (which is maintained into adulthood) and show no motor deficits up to 1 year of age. However, at 6 weeks they begin to develop axonal swellings with the accumulation of organelles, especially in the optic nerve (suggesting defects in axonal transport). As swellings increase, secondary demyelination occurs, and, at 1-year old, axon degeneration becomes prominent.

Similar observations have been made in the PNS. Certain patients carrying mutations in the myelin protein zero (MPZ) gene, the major component of PNS myelin, develop an axonal form of neuropathy in the absence of any detectable myelin defects. Misu et al. (2000) reported seven Japanese families with an axonal form of CMT disease characterized by late-onset, marked sensory impairment and muscle atrophy, but with preserved sensory and motor nerve conduction velocities. Another group, Bienfait et al. (2006), identified a different MPZ mutation also causing an axonal CMT which they found, by computational modeling, should not affect the structure of the protein. This explains the preserved myelin stability and suggests that the pathology is caused by a myelin-independent mechanism.

Rodent models with disrupted SC metabolism provided further insight into the potential role of SC in axonal support. Prominent examples are the SC knockouts of LKB1 (Beirowski et al., 2014), TFAM (Viader et al., 2011, 2013) and Cox10 (Fünfschilling et al., 2012). Common to all three models is the adaptability of SC to metabolic constraints. SC seem to be able to survive and, to some extent, exert their functions in the absence of mitochondrial respiration and under disturbed metabolic balance, possibly by switching to aerobic glycolysis. LKB1 and TFAM mutant SC can produce normal myelin by early adulthood (P30) but this does not prevent early loss of small unmyelinated axons, which progresses to loss of myelinated fibers, with (for TFAM mutants) or without previous demyelination (in the case of LKB1-deficiency). LKB1 mutants show reduced lipid and protein synthesis (due to reduced mTORC1 activity), while TFAM mutants show reduced lipid synthesis, increased oxidation and accumulation of toxic lipid species. As a consequence, it is possible, that at the molecular level, the interactions between neurons and glia in these models are not as normal as they seem and that the abnormal protein and lipid make-up of the mutant SC may induce stress in the neighboring neurons, either by the lack of beneficial factors or presence of toxic ones. The Cox10 mutant, on the other hand, presents a much stronger phenotype in the PNS, with defects in sorting and myelination, abnormal myelin, and reduced number of myelinated axons, suggesting Cox10 is necessary for early SC functions. In this model, however, Cox10-deficient oligodendrocytes were able to myelinate (due to the presence of healthy mitochondria produced before Cre-recombination) and maintain myelinated axons despite impaired oxidative metabolism. Increased production of lactate was detected in the CNS, suggesting increased aerobic glycolysis, but this was not demonstrated for SC. Interestingly, increased lactate release was observed in LKB1-deficient SC and is thought to be a way to metabolically support distressed neurons, since inhibition of lactate release exacerbated axonal loss. It would be relevant to verify if increased lactate production occurs in different models of SC mitochondrial deficiency and if exogenous supplementation can have a neuroprotective effect.

## Mechanisms Potentially Implicated in the Metabolic Coupling Between SC and Axons

In large animals, long peripheral axons can reach lengths of several meters. Such distances between neuronal soma and nerve endings make it inefficient to transport nutrients and maintain axonal function. Additionally, myelin itself acts as a barrier to diffusion of molecules, and nodes of Ranvier are too small to account for sufficient uptake of extracellular or blood-borne glucose (Nave, 2010). Nevertheless, SC seem well-positioned to act as intermediaries between axons and the extracellular milieu. After myelination is complete, non-compact myelin regions and cytoplasmic channels (such as Schmidt-Lanterman incisures) are left behind, creating a potential route of transport for metabolites from the abaxonal SC cytoplasm to the periaxonal space. SC express glucose transporters (GLUT) 1 and 3 (Magnani et al., 1996), MCT1, 2 and 4 (Domènech-Estévez et al., 2015) and connexins (Altevogt et al., 2002), making them well-equipped to import and export high-energy substrates between their cytoplasm, the extracellular environment, and underlying axons.

Mice lacking one copy of MCT1 gene present normal myelin but show impaired axon regeneration after sciatic nerve crush injury (Morrison et al., 2015) while MCT1-depleted SC have reduced ability to phagocyte myelin debris, promote axon re-growth and remyelinate, which are highly demanding metabolic tasks (Zochodne, 2012). This could be due to reduced energy levels in SC caused by the inability to import lactate for oxidative phosphorylation, or to imbalanced pH and redox status due to reduced export of glycolysis by-products. A recently published conditional knockout of MCT1 in SC (Jha et al., 2020) shows normal development and myelination of peripheral nerves, with progressive thinning of myelin in sensory fibers accompanied by reduced sensory nerve conduction and mechanical sensitivity. These results suggest that altered transport of energy-rich metabolites impairs SC function and that sensory neurons are more susceptible to metabolic imbalances, a common feature of peripheral neuropathies.

Other components can be found at the axon-SC unit (Samara et al., 2013) including receptors for neurotransmitters [NMDA receptors (Campana et al., 2017) and metabotropic glutamate receptors (Saitoh et al., 2016)] expressed by SC. Their presence suggests that SC may be able to detect axonal activity and that neuron-SC communication may be involved in activity-dependent metabolic support. In the CNS, oligodendrocytes enhance their glucose uptake upon stimulation with NMDA (Saab et al., 2016). Oligodendrocytes lacking NMDAR subunit NR1 show lower levels of GLUT and are unable to support axonal function after an ischemic insult when glucose and oxygen are provided. However, perfusion with lactate and oxygen after ischemia recovers axonal function to control levels, showing that lactate is readily used by neurons as opposed to glucose. In the PNS, SC can also respond to glutamate signaling *via* NMDAR (Campana et al., 2017). Activation of NMDAR *in vitro* leads to a signaling cascade involving PI3K, Akt, and ERK pathways, which are involved in metabolic regulation. Activation of ERK also occurs *in vivo* in response to glutamate or NMDA injection in the sciatic nerve, further corroborating the idea that SC can respond to axonal cues.

During development, bi-directional communication between axons and SC control the early stages of SC proliferation, axonal contact, sorting and myelination (reviewed in Fledrich et al., 2019; Wilson et al., 2020). However, it is not clear if SC play a role in providing energy substrates directly to axons at this stage. It is possible that in a smaller, less organized developing nerve, blood-borne metabolites might diffuse more easily and be taken up directly by neurons. The aforementioned Cox10 cKO (Fünfschilling et al., 2012) presents extensive and lasting defects in radial sorting and myelination, while the delay observed in SC-specific LKB1 mutant (Beirowski et al., 2014) normalizes by P30. It is impossible, at this moment, to discern the impact that SC-specific metabolic defects may have on axons during a developmental stage that is itself metabolically demanding on SC as it would be necessary to block neuronal access to SC metabolites without preventing normal SC development.

## Metabolic Interaction Between SC and Axons in the Context of Disease and Therapy

In diabetic neuropathy (DN, Figure 1), one of the leading causes of peripheral nerve disease, unmyelinated C fibers, and small myelinated axons are the first to be affected and their degeneration and regeneration cause sensory symptoms such as pain and sensitivity to non-noxious stimuli, as well as the absence of sensation (Said et al., 1983; Malik et al., 2005; Zenker et al., 2013; Feldman et al., 2017). In neuropathies characterized by loss of small unmyelinated fibers, skin denervation is one of the initial signs of pathology and correlates with disease severity (Ebenezer et al., 2007). These axons are in close contact with non-myelinating SC and wrapped in Remak bundles. The absence of protective myelin and the small cytoplasmic volume could render these axons more susceptible to the metabolic imbalance characteristic of diabetes (activation of the polyol pathway and increased ROS, for example, Feldman et al., 2017). Despite the considerable advancements in knowledge of myelinating SC metabolism and relationship with axons, the field is still lacking efficient approaches to study non-myelinating Remak cells. The development of an inducible conditional Cre driver that sparsely labels nervous system cells, including nmSC (Sapkota and Dougherty, 2019), is an ingenious strategy. However, caution must be exercised when using such a model for conditional knockout studies as only 15% of nmSC are recombined and other cell types might be affected in non-negligible amounts, such as neurons and astrocytes, and therefore the co-expression of a reporter gene is advisable. The metabolic interaction between SC and axons could potentially be exploited for therapeutic applications. In demyelinating conditions such as CMT1A, improving myelination *per se* can lead to better axonal support. This makes it difficult to reveal the potential beneficial role of these approaches on the capacity of SC to support axons. A combination of baclofen, naltrexone, and sorbitol named PXT3003 showed in animal studies the ability to reduce mRNA levels of Pmp22 and improved downstream signaling pathways, effectively delaying disease onset (Prukop et al., 2019). Fledrich et al. (2018) reported the positive effect of dietary supplementation with phosphatidylcholine and phosphatidylethanolamine on myelination in a rat model of CMT1A, with improvement at the level of grip strength, nerve conduction velocity and the number of myelinated axons. Similarly, Sahenk et al. (2018) observed improved myelination and nerve conduction outcomes in Trembler-J mice treated with pyruvate alone or in combination with virally-administered neurotrophin 3 gene therapy.

In line with the proposed role of glial metabolic support in axonal function, one possible therapy to treat the diseased PNS is the use of a ketogenic diet. This diet consists of the elimination of carbohydrates and their replacement with fats while maintaining an adequate supply of proteins. A ketogenic diet drives the production of ketone bodies such as acetoacetate by the liver, which can be readily utilized by neurons, as they are transported by MCTs and feed directly into the tricarboxylic acid cycle in the mitochondria for energy production, similar to lactate and pyruvate. In the CNS, a ketogenic diet approach has shown marked improvement in a model of Pelizaeus–Merzbacher disease (Stumpf et al., 2019) with an increased number of oligodendrocytes, myelinated axons and the concomitant decrease in mean g-ratio. Mitochondrial abnormalities were also improved, even in unmyelinated axons, suggesting a ketogenic diet can improve axonal function independently of myelin recovery. Ketone bodies, due to their small molecular size and chemical properties can cross the blood-brain barrier, unlike larger lipids and cholesterol. For this reason, they represent an interesting therapeutic strategy, as the development of drugs targeting neurodegenerative diseases stalls. Administering a ketogenic diet to mice leads to reduced production of reactive oxygen species in sciatic nerve mitochondria (Cooper et al., 2018b) and rescues mechanical allodynia caused by a high-fat diet (containing 54% fat and 24% carbohydrates; Cooper et al., 2018a). Ketones also promote axonal growth *in vitro* from dorsal root ganglion neurons. As an intervention, switching to a ketogenic diet after 8 weeks on a high-fat diet did not reverse obesity or markers of prediabetes but it did increase skin innervation and reversed mechanical allodynia, hallmarks of diabetic neuropathy. This suggests that the effects of a ketogenic diet on overall metabolism are different from the effects on peripheral nerves. These observations are quite encouraging but it is yet to be determined if a ketogenic diet can be beneficial for other types of peripheral neuropathy, such as axonal forms of CMT.

## Conclusion

With all recent development in the field of neuron-glia interactions, the PNS is still a largely unexplored territory. Peripheral neuropathies affect a large proportion of the world’s population and implicate costs in both treatment and loss of productivity. Nevertheless, they are a heterogeneous group of conditions that will certainly warrant different preventative and therapeutic approaches. The data accumulated so far indicate that interactions between SC and axons may represent druggable targets for therapeutic applications. Also, their further characterization may yield new biomarkers allowing the detection and prognosis of further damage to SC and/or axons.

## Author Contributions

FB wrote the first draft of the manuscript. Both authors contributed to manuscript revisions, read and approved the submitted version.

## Conflict of Interest

The authors declare that the research was conducted in the absence of any commercial or financial relationships that could be construed as a potential conflict of interest.
